# Molecular Study of Dietary Heptadecane for the Anti-Inflammatory Modulation of NF-kB in the Aged Kidney

**DOI:** 10.1371/journal.pone.0059316

**Published:** 2013-03-26

**Authors:** Dae Hyun Kim, Min Hi Park, Yeon Ja Choi, Ki Wung Chung, Chan Hum Park, Eun Ji Jang, Hye Jin An, Byung Pal Yu, Hae Young Chung

**Affiliations:** 1 Department of Pharmacy, College of Pharmacy, Aging Tissue Bank, Pusan National University, Gumjung-gu, Busan, Republic of Korea; 2 Department of Physiology, The University of Texas Health Science Center at San Antonio, San Antonio, Texas, United States of America; Chang Gung University, Taiwan

## Abstract

Heptadecane is a volatile component of Spirulina platensis, and blocks the *de novo* synthesis of fatty acids and ameliorates several oxidative stress-related diseases. In a redox state disrupted by oxidative stress, pro-inflammatory genes are upregulated by the activation of NF-kB via diverse kinases. Thus, the search and characterization of new substances that modulate NF-kB are lively research topics. In the present study, heptadecane was examined in terms of its ability to suppress inflammatory NF-kB activation via redox-related NIK/IKK and MAPKs pathway in aged rats. In the first part of the study, Fischer 344 rats, aged 9 and 20 months, were administered on average approximately 20 or 40 mg/Kg body weight over 10 days. The potency of heptadecane was investigated by examining its ability to suppress the gene expressions of COX-2 and iNOS (both NF-κB-related genes) and reactive species (RS) production in aged kidney tissue. In the second part of the study, YPEN-1 cells (an endothelial cell line) were used to explore the molecular mechanism underlying the anti-inflammatory effect of heptadecane by examining its modulation of NF-kB and NF-kB signal pathway. Results showed that heptadecane exhibited a potent anti-oxidative effect by protecting YPEN-1 cells from tert-butylhydroperoxide induced oxidative stress. Further molecular investigations revealed that heptadecane attenuated RS-induced NF-kB via the NIK/IKK and MAPKs pathways in YPEN-1 cells and aged kidney tissues. Based on these results, we conclude that heptadecane suppresses age-related increases in pro-inflammatory gene expressions by reducing NF-kB activity by upregulating the NIK/IKK and MAPKs pathways induced by RS. These findings provide molecular insight of the mechanisms by which heptadecane exerts its antiinflammatory effect in aged kidney tissues. We conclude that heptadecane suppresses age-related increases in pro-inflammatory gene expressions then travel upstream set by step by reducing NF-kB activity by downregulating the NIK/IKK and MAPKs pathways induced by RS.

## Introduction

Heptadecane is major component of Spirulina platensis [1], which contains high levels of proteins, amino acids, vitamins, beta-carotene, and other pigments [2], and it has been shown that the strong antioxidative effects of have therapeutic benefit in a rat renal disease model [3], and that it inhibits the proliferation of human liver cancer cells [4]. Moreover, heptadecane was found to almost completely block the *de novo* synthesis of fatty acids *in vitro* [5]. However, despite the amount of information available on the biological activities of heptadecane, little is known of the molecular mechanisms by which heptadecane affects various intracellular activities.

Aging is an inevitable consequence of processes characterized by age-dependent physiological functional decline. The basic cellular and biochemical features of the aging process are complex, but the most widely accepted theory is that aging is caused by oxidative stress [6]. The oxidative stress hypothesis of aging describes the characteristic changes of the aging process as being the net effect of redox imbalances between oxidative stress and anti-oxidative mechanisms, which include oxidative alterations in DNA, protein, and other cellular components, including antioxidant defense systems, which accumulate causing functional deficits and many age-related degenerative diseases [6].

Oxidative stress causes redox imbalances of various reactive oxygen species (ROS), such as, superoxide (•O_2_^−^), hydrogen peroxide (H_2_O_2_), and the hydroxyl radical (•OH), and of various reactive nitrogen species (RNS), such as, nitric oxide (NO) and peroxynitrite (ONOO^-^). Among the several well-known hypotheses of aging, the oxidative stress hypothesis currently offers the best mechanistic description of the aging process and of age-related chronic disease processes [6]. Recent research reports provide evidence that oxidative processes are a major factor in the activation of redox-sensitive inflammatory processes, and that they act as a bridge between the normal aging process and age-related chronic diseases [7], [8].

Furthermore, the disruption of the intricate redox balance regulated in part by oxidative stress, influences age-related alterations in signaling transduction and gene regulation. As redox potential is modulated by oxidative stress, various cellular processes related to signaling and gene expression can be inhibited or activated in response to oxidative changes in the intracellular environment [6]. In particular, the effects of redox imbalance caused by oxidative stress is capable of greatly affecting redox-sensitive gene regulation, including the upregulation of genes whose expressions are positively associated with pro-inflammatory NF-kB (nuclear factor-kB) during aging [7], [8], [9], [10].

It has been shown that the activation of redox-sensitive NF-kB plays a pivotal role in the cellular signaling mechanism for oxidative stress-induced inflammation during aging [7], [8]. The key features of NF-kB are it is redox-responsive and a critical regulator of a variety of pro-inflammatory genes, such as, cytokines and chemokines [10], [11], [12]. In addition, the regulation of the nuclear translocation of NF-kB is also redox sensitive as demonstrated by the required phosphorylation of its p65 subunit, which is required for the transcriptional activation [1], [13], [14]. Diverse kinases are reported to phosphorylate specific amino-terminal serine residues at IκB in NF-kB [15]. The majority of these studies support the upstream activations of IkB kinase (IKK), IKKα, and IKKβ, which are phosphorylated by NF-kB-inducing kinase (NIK) [16]. Also, p38 mitogen activated protein kinase (p38MAPK), extracellular signal-regulated kinase (ERK), and c-jun-N-terminal kinase (JNK) constitute an additional level of gene regulation by NF-kB [17], [18]. Each of these MAPK subfamilies is activated by specific upstream mitogen-activated protein kinase kinases (MKKs), which dually phosphorylate MAPKs on threonine and tyrosine residues separated by an intervening amino acid unique to specific MAPK subfamilies [19], [20].

For the current, the kidney was selected for its sensitivity to oxidative stress, vulnerability to aging, and the responsiveness to age-related inflammatory responses, all which make the kidney as one of most suitable organs of the choice to determine the biological function of heptadecane and to examine its anti-oxidative and anti-inflammatory potentials. We fed heptadecane to aging rats, and performed *in vitro* studies on an endothelial cell line. Here, we report the suppressive effect of heptadecane on NF-kB and on the ability of heptadecane to reduce age-related oxidative stress and to modulate the NIK/IKK and MAPKs cascades.

## Results

### Modulation of Age-related NF-kB Activation by Heptadecane

**Figure 1:**
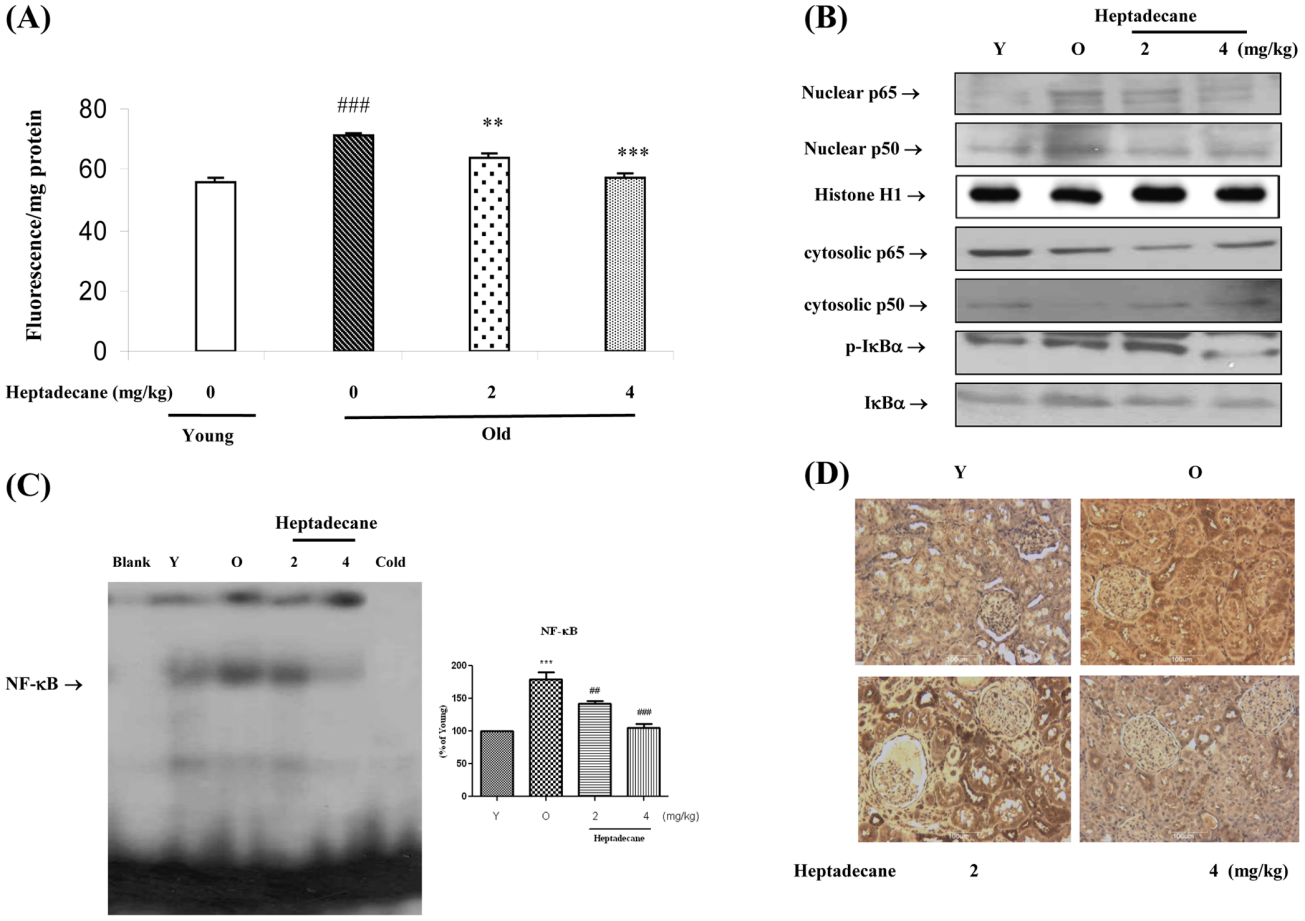
Heptadecane suppressed age-related increases in NF-kB activity. (A) RS generation in aged rat was determined by observing the effects of age and heptadecane using the DCFHDA method and kidney homogenates. The values shown are means ± SEs for 5 rats. Young = 9-month-old baicalin untreated rats; Aged = 20-month-old, heptadecane untreated rats. Values are means ± SEs for 5 rats. ^###^p<0.001 vs. Young rats; **p<0.01 vs. age-matched rats; ***p<0.001 vs. age-matched rats by one factor ANOVA. Western blot was performed to detect (B) nuclear p50 and p65 protein levels in nuclear extracts (30 µg protein) and age-related IkBα, IkBβ, and IkBα phosphorylation degradations in cytoplasmic extracts (40 µg protein) in young, aged, and aged rats fed heptadecane. (C) EMSA method was used to compare the nuclear NF-kB binding activities of aged rat fed heptadecane and aged counterparts. One representative result of three experiments that yielded similar results is shown. Young rats (9 months of age) and aged (20 months of age) were utilized. Heptadecane was fed to the aged rat at 2 mg or 4 mg/Kg per day for 10 days. Statistical significance: results of one-factor ANOVA: ***p<0.001 vs. young rat; ^##^p<0.01, ^###^p<0.001 vs. old non-heptadecane-fed rats, respectively. (D) Immunoreactivity was determined by NF-kB (p65) in renal tissue of age and heptadecane (Immunohistochemistry, ×100).

To assess overall age-related oxidative status and its modulation by heptadecane, total RS was measured in kidney homogenates using a DCFDA probe. The results showed that RS level increased with age and that this increase was significantly suppressed by treatment with high-dose heptadecane (Fig. 1A). To determine whether or not NF-kB activation is increased during aging, we examined nuclear protein levels by Western blotting using p65- and p50-specific polyclonal antibodies. The results shown in Fig. 1B clearly reveal that the nuclear translocation of NF-kB was significantly greater in aged rats, but that aged heptadecane-fed rats showed dose-dependently lower levels of NF-kB. Furthermore, as shown in Fig. 1B, aged heptadecane-fed rats showed higher levels of IkBα and IkBβ proteins in cytoplasmic extracts than aged rats, while Thus, these findings indicate that NF-kB translocation during aging is probably elicited by age-related increases in the degradations of IkBα and IkBβ.

To verify NF-kB DNA-binding, EMSA was carried out using nuclear proteins isolated from young, aged, and aged rats fed heptadecane. The results shown in Fig. 1C indicate that the binding activity of NF-kB was upregulated during aging, and that heptadecane suppressed this upregulation. Additionally, we performed immunohistochemistry analysis on aged rat renal tissue for the immunoreactivity using anti-NF-kB (p65) antibody. As shown in Fig. 1D, intensive NF-kB increased compared with that of tubular cells of young controls, indicating the suppression of the over expression of NF-kB by heptadecane.

### Suppression of NIK/IKK and of MAPK Activation by Heptadecane during Aging

**Figure 2:**
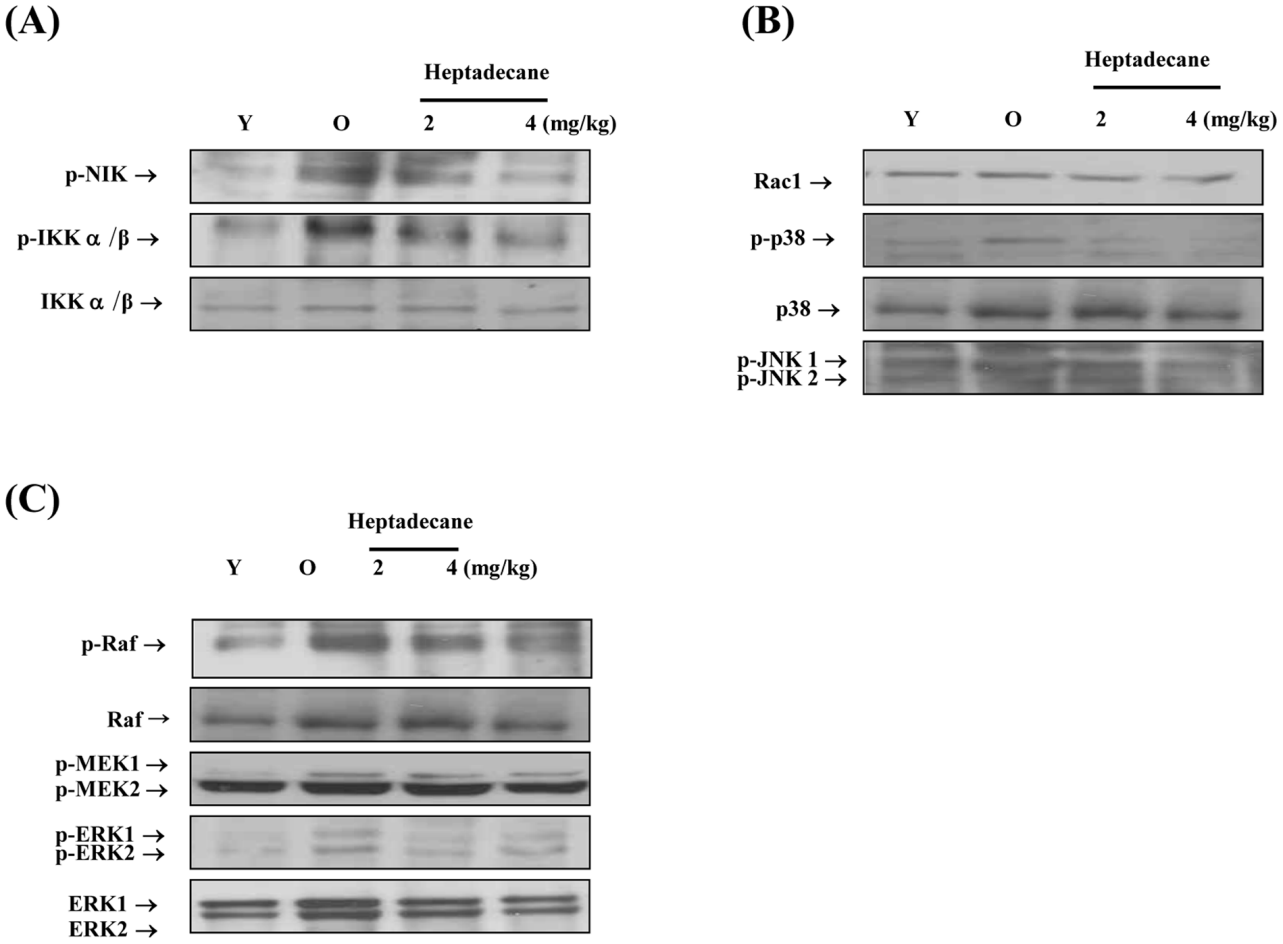
Heptadecane suppressed the age-related activations of NIK/IKK and MAPKs. Western blot on renal cytoplasmic extracts (40 µg protein) from young, aged, and aged rats fed heptadecane. (A) Phosphorylations of P38 and JNK are designated p-p38 and p-JNK1/2. (B) Phosphorylations of NIK and IKK are designated p-NIK and p-IKKα/β. (C) Phosphorylations of MEK1/2 and ERK1/2 were detected using antibodies of p-MEK1/2 and p-ERK1/2. One representative blot of each protein from three experiments that yielded similar results is shown. Young rats (9 months of age) and aged (20 months of age) were utilized. Heptadecane was fed to aged rats at 2 mg or 4 mg/Kg per day for 10 days.

We investigated whether heptadecane can regulate the phosphorylations of the NIK/IKK and MAPKs pathways which lead to NF-kB activation during aging. The results obtained showed that the phosphorylations of NIK/IKK and MAPKs were significantly increased in aged rats; however, aged, heptadecane-fed rats showed dose dependent lower levels than their aged, non-heptadecane-fed counterparts (Fig. 2).

These results show that heptadecane supplementation significantly inhibited the oxidative stress-induced phosphorylations of NIK/IKK and MAPKs during aging, and that heptadecane blocks the phosphorylation and degradation of IkB, which elicit the nuclear translocation of NF-kB.

### Inhibition of NF-kB-responsive Gene Expression by Heptadecane

**Figure 3:**
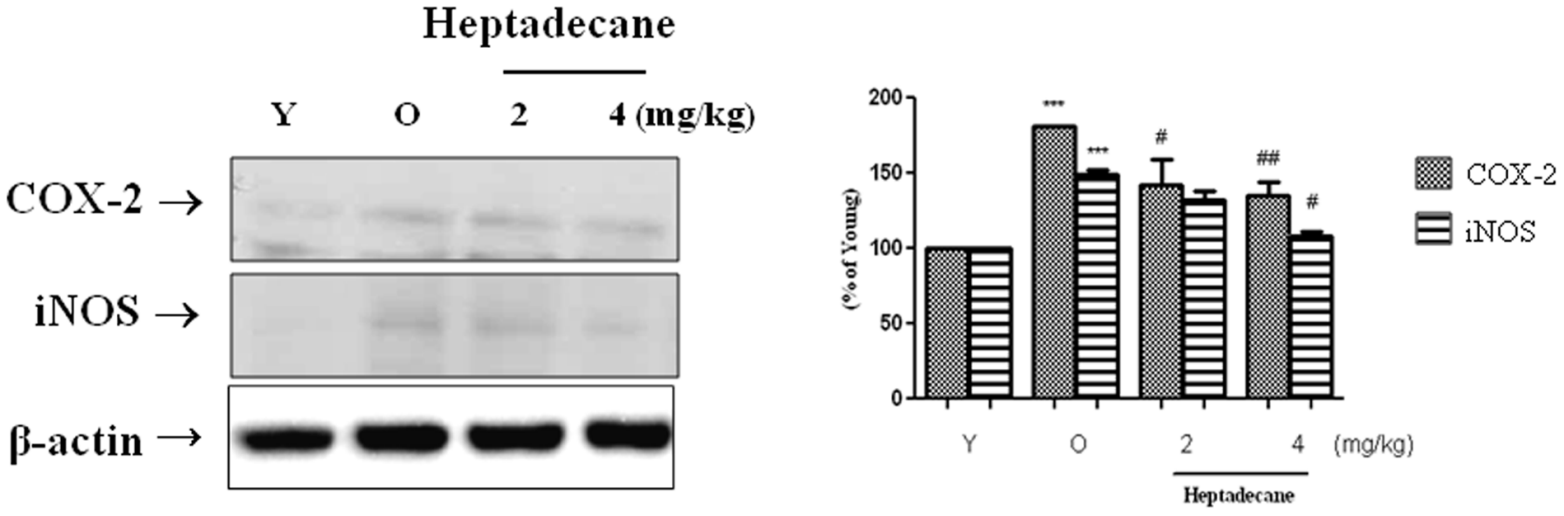
Heptadecane suppressed expressions of NF-kB-dependent genes in aged rats. Western blot was performed to detect renal COX-2 and iNOS levels in cytoplasmic extracts (40 µg protein) from young, aged, and aged rats fed heptadecane. One representative blot of each protein is shown from three experiments that yielded similar results, respectively. Young rats (9 months of age) and aged (20 months of age) were utilized. Heptadecane was fed to aged rats at 2 mg or 4 mg/Kg per day for 10 days. Statistical significance: results of one-factor ANOVA: ***p<0.001 vs. young rat; ^#^p<0.05, ^##^p<0.01 vs. old non-heptadecane-fed rats, respectively.

Above all, our findings data indicate that NF-kB activation is involved in age-related oxidative stress (Fig. 3), which leads to age-related inflammation and that this age-related NF-kB activation is strongly inhibited by heptadecane. In addition, we examined the gene expressions of iNOS and COX-2 (NF-kB-dependent genes), which both have a kB-site located in their promoter regions. Our results showed that the protein levels of these two genes were positively related to NF-kB activity and that heptadecane down-regulated their expressions.

### Effect of Heptadecane on Oxidative Stress

The oxidant, t-BHP, is a known to induce oxidative stress [21]. To determine the defensive effect of heptadecane against oxidative stress, t-BHP was used to induce oxidative stress in YPEN-1 cells. RS generation in YPEN-1 cells using DCFDA, which is oxidized by RS to fluorescent DCF. YPEN-1 cells treated with 10 µM t-BHP displayed fluorescent intensity before incorporation with DCFDA.

**Figure 4:**
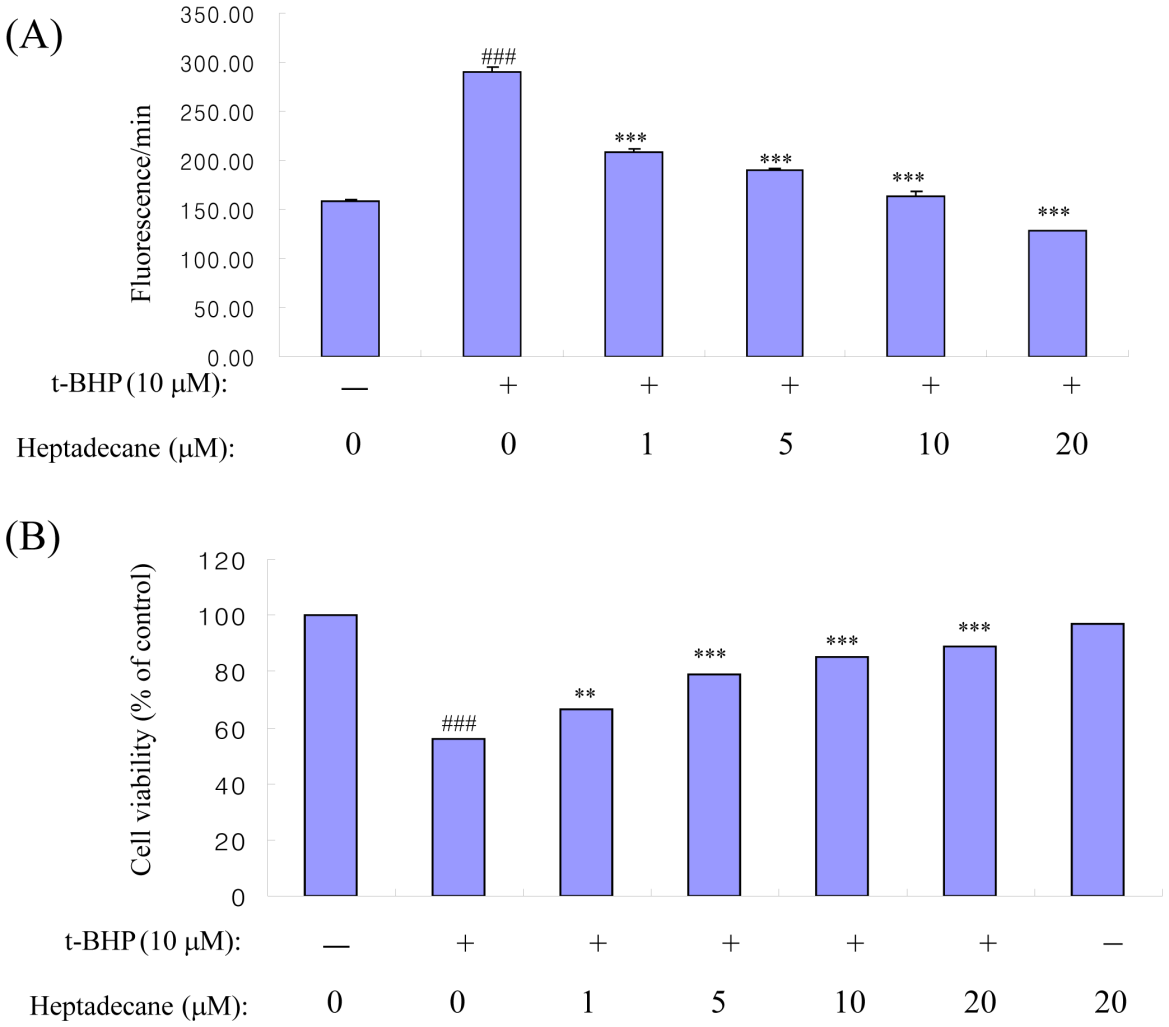
Heptadecane protected YPEN-1 cells from t-BHP-induced oxidative stress and cytotoxicity. (A) Cells were treated with or without/heptadecane at concentrations of 1, 5, 10, and 20 µM for 1 hr and then with vehicle or 10 µM t-BHP. (B) Cells were treated with or without heptadecane at concentrations of 1, 5, 10 and 20 µM for 1 hr and then with t-BHP (10 µM) for 6 hr. Viable cell numbers were determined using an MTT assay. ^##^p<0.01 vs. vehicle treated control group; **p<0.01, ***p<0.001 vs. the 10 µM t-BHP group by one-factor ANOVA.

Furthermore, intracellular RS formation was significantly reduced when heptadecane (1, 5, 10, or 20 µM) was present in medium (Fig. 4A). To determine the cytoprotective effect of heptadecane on oxidative stress-induced cytotoxicity by t-BHP, cell viabilities were measured using an MTT assay, and cells pre-treated with heptadecane these concentrations (Fig. 4B) demonstrated a dose-dependent cytoprotective effect.

### Suppressive Action of Heptadecane on Oxidative Stress-induced NF-kB in YPEN-1

**Figure 5:**
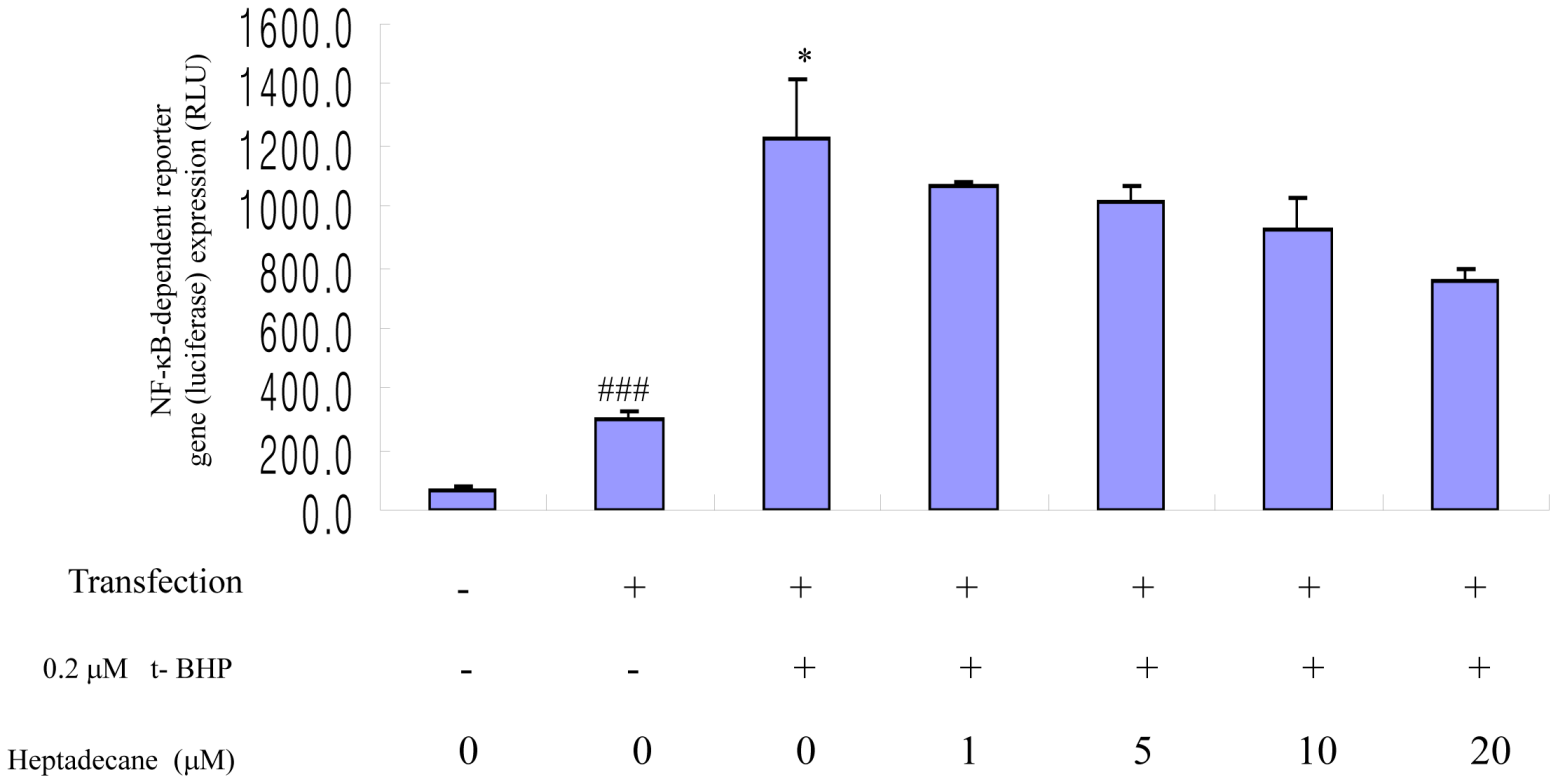
Heptadecane inhibited oxidative stress-induced NF-kB-dependent reporter gene (luciferase) expression. YPEN-1 cells were transiently transfected with a NF-kB-containing plasmid linked to the luciferase gene, then pre-incubation with heptadecane (1, 5, 10 µM and 20 µM) for 1 hr and co-treated with t-BHP for 5 hr. Results are presented in relative luminescence units (RLU). One-way analysis of variance (ANOVA) was used to determine the significances of NF-kB-dependent luciferase activity differences between untreated controls and treated groups: ^##^p<0.01 vs. vector control, *p<0.05 vs. treated 0.2 µM t-BHP group, respectively.

To confirm the effect of heptadecane on oxidative stress-induced NF-kB activation in YPEN-1, cells were transiently transfected with plasmid containing the NF-kB consensus sequence and luciferase reporter luciferase activity was then detected by adding t-BHP in the absence or presence of heptadecane (Fig. 5). NF-kB luciferase activity increased by 4-fold compared to transfected cell exposure to 0.2 µM t-BHP for 6 h, and heptadecane exhibited a dose-dependent ability to decrease NF-kB luciferase activity. Overall, this result indicates that NF-kB activation is involved in t-BHP-induced oxidative stress, and that this activation is inhibited by heptadecane.

### Suppressive Effects of Heptadecane on NF-kB Target Gene Expressions via IKK/p38/ERK/JNK

**Figure 6:**
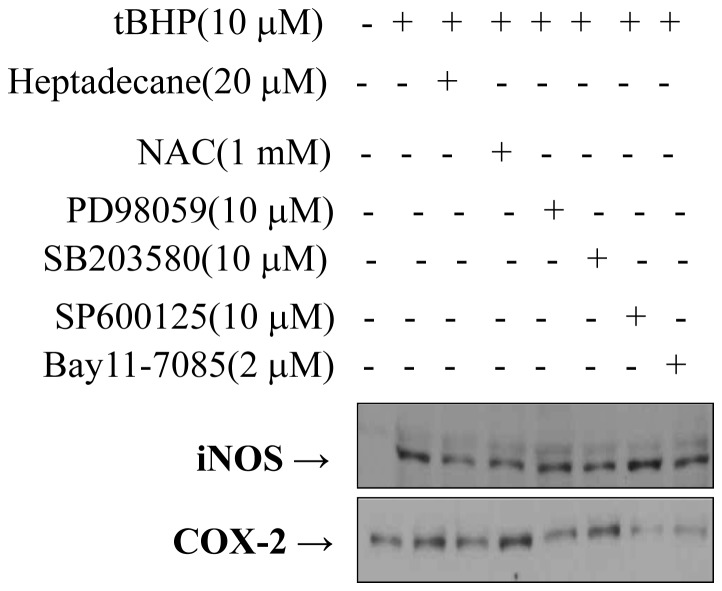
Heptadecane attenuated NF-kB-responsive COX-2 and iNOS via NIK/IKK and MAPKs. YPEN-1 cells were grown to 80% confluent in 100 mm dishes in DMEM. Cells were pre-treated (1 hr) with heptadecane (20 µM) and inhibitors and then stimulated with 10 µM t-BHP. After stimulation with t-BHP in the absence (−) or presence (+) of heptadecane and each kinase inhibitor, COX-2 and iNOS gene expressions were determined in cell extracts.

COX-2 and iNOS are NF-kB responsive genes and are regulated by various kinases that cause the translocation of NF-kB [22]. To confirm the mechanism of the NF-kB pathway, YPEN-1 cells were pretreated with heptadecane, IKK inhibitor (BAY11-7085), p38 inhibitor (SB203580), ERK inhibitor (PD098059), JNK inhibitor (SP600125), or RS inhibitor (NAC) for 1 hr. After these cells were then incubated with 10 µM t-BHP for 6 hr, COX-2 and iNOS expressions were assessed. As shown in Fig. 6, t-BHP induced COX-2 and iNOS were suppressed by heptadecane and kinase inhibitors, suggesting that heptadecane down-regulated COX-2 and iNOS through these kinases.

**Figure 7:**
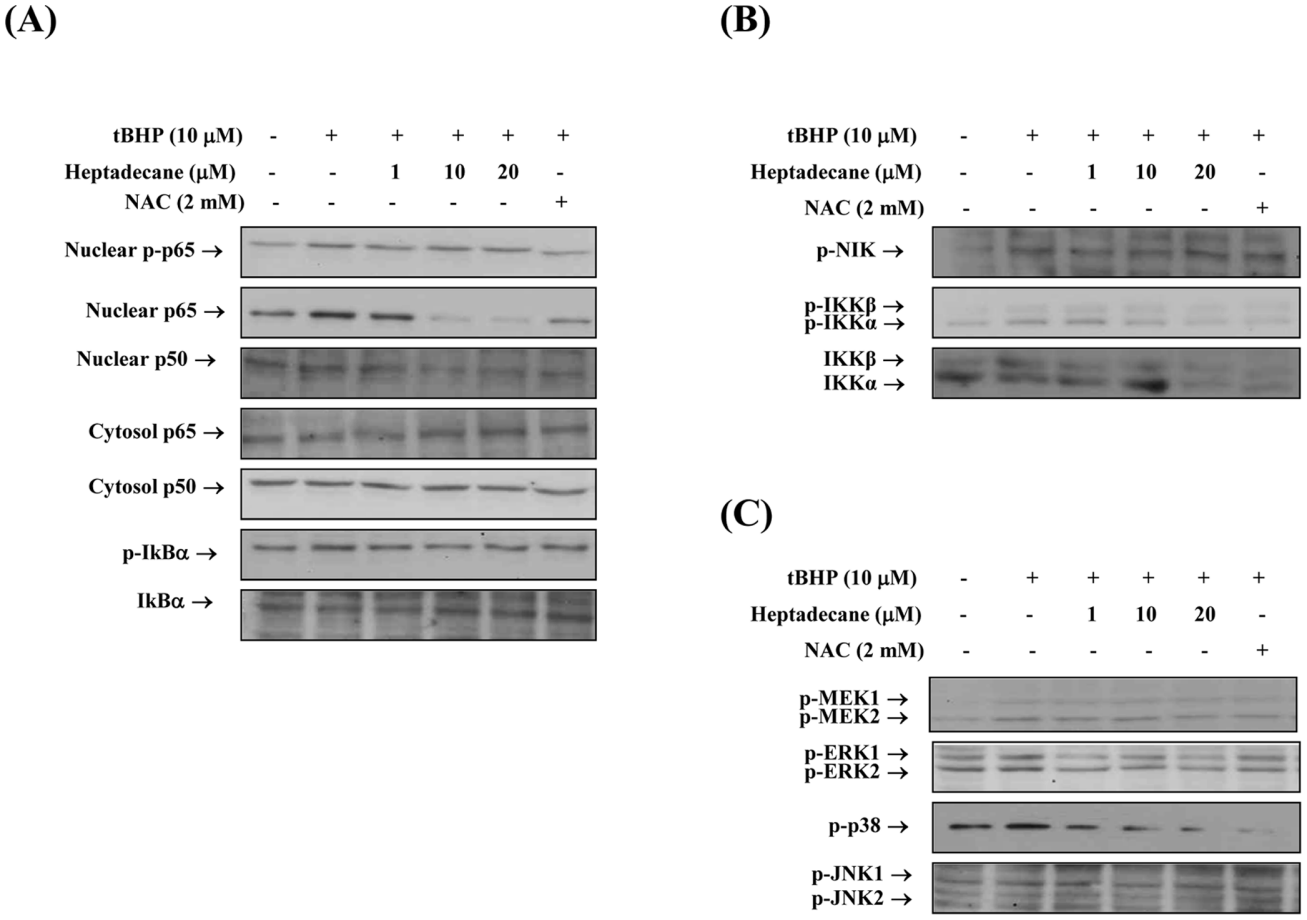
The regulation of NF-kB by heptadecane via NIK/IKK and MAPKs induced by oxidative stress. YPEN-1 cells were grown until 80% confluent in 100 mm dishes in DMEM medium. Cells were pre-treated (1 hr) with heptadecane (1, 10, or 20 µM) or NAC (2 mM), then stimulated with 10 µM t-BHP. (A) After stimulation with t-BHP (1 hr for NF-kB) in the absence (-) or presence (+) of heptadecane (1, 10, or 20 µM), cells lysed and total nuclear and cytosolic proteins were extracted. Western blot was performed for p50, p65 and IκB. (B) After stimulation with t-BHP (10 min for phosphorylated NIK and 20 min for phosphorylated IKK) in the absence (-) or presence (+) of heptadecane (1, 10 or 20 µM), cells were lysed and p-NIK and p-IKKα/β levels were determined. (C) After stimulation with t-BHP (10 min for phosphorylated MEK1/2 and 20 min for phosphorylated MAPKs) in the absence (-) or presence (+) of heptadecane (1, 10, or 20 µM), cells were lysed and p-MEK1/2, p-ERK1/2, p-p38, and p-JNK levels were determined. One representative experimental blot of each protein is shown from three experiments that yielded similar results.

In addition, we tested the effects of heptadecane on the phosphorylations of IKKα/β, ERK1/2, p38, and JNK, which can activate NF-kB (Fig. 7A) under conditions of oxidative stress, and we examined the more upper step of NIK and MEK kinases (Fig. 7B and C). We found that heptadecane significantly inhibited the phosphorylations of NIK/IKK and MAPKs caused by t-BHP-induced oxidative stress. These results suggest that heptadecane prevents NF-kB nuclear translocation via NIK/IKK and MAPKs.

## Discussion

This study was undertaken to determine the biological function of heptadecane and to examine its anti-oxidative and anti-inflammatory potentials. Accordingly, heptadecane was fed to aging rats, and in vitro studies were performed on an endothelial cell line. Our results show that heptadecane suppresses age-related increases in pro-inflammatory gene expressions by reducing NF-kB activity by upregulating the NIK/IKK and MAPKs pathways induced by RS, and provide insight of the mechanisms by which heptadecane exerts its anti-inflammatory effects in aged kidney tissues.

Heptadecane is major component of Spirulina, which contains high levels of proteins, amino acids, vitamins, beta-carotene, and other pigments [2]. Furthermore, it has been reported that antioxidative effects of heptadecane have therapeutic potential in the context of renal diseases in a rat model [3] and have inhibitory effects on the proliferation of human liver cancer cells [4].

Oxidative stress is considered one of the major contributors to age-associated redox imbalance and NF-kB activation [23]. Under increased oxidative conditions, such as, during aging, redox deregulation occurs, and as shown by the present study, increased RS (Fig. 1) results in higher redox potentials and leads to cellular damage. Because elevated RS levels deplete intracellular thiol groups, a state of cumulative cellular damage ensues [24]. The association between redox potential and NF-kB activation is central to the age-related inflammatory process because of the sensitivity of NF-kB to oxidative stress [8], [9], [10]. Redox sensitive NF-kB is a multi-subunit eukaryotic transcription factor that consists of either homo- or heterodimers of various members of the Rel family, such as, p50, p52, p65 (RelA), c-Rel and RelB [25]. When the redox system is disrupted, IkBα is phosphorylated at serine residues, ubiquitinated at lysine residues, and degraded through the proteosomal pathway, which exposes the nuclear localization signals on p50-p65 heterodimer. p65 then undergoes phosphorylation, leading to the nuclear translocation of NF-kB and its binding to a specific DNA sequence, which in turn results in the transcriptions of genes, such as, TNF, interleukins, ICAM-1, VCAM-1, iNOS, and COX-2 [26], [27].

NF-kB activation has been suggested to be mediated by two distinct redox-related signaling pathways. First, the NF-kB translocation dependent pathway involves IKKα/β dependent IkBα phosphorylation and degradation [28], [29]. These are key regulatory steps that dictate redox-sensitive NF-kB activation, and thus, the focus of this study was on the status of NIK/IKK phosphorylation and on its modulation during aging. Second, the phosphorylation of ERK, p38 MAPK, or JNK leads to NF-kB translocation [30], [31]. MAPKs are important redox-sensitive mediators, which with other proteins transduce extracellular cues to intracellular responses through MEK [32]. Furthermore, some evidence shows that IKK and MAPKs are induced by oxidative stress and can be regulated by strong antioxidants (e.g., resveratrol, epigallocatechin gallate, or vitamin C [33], [34], [35].

**Figure 8:**
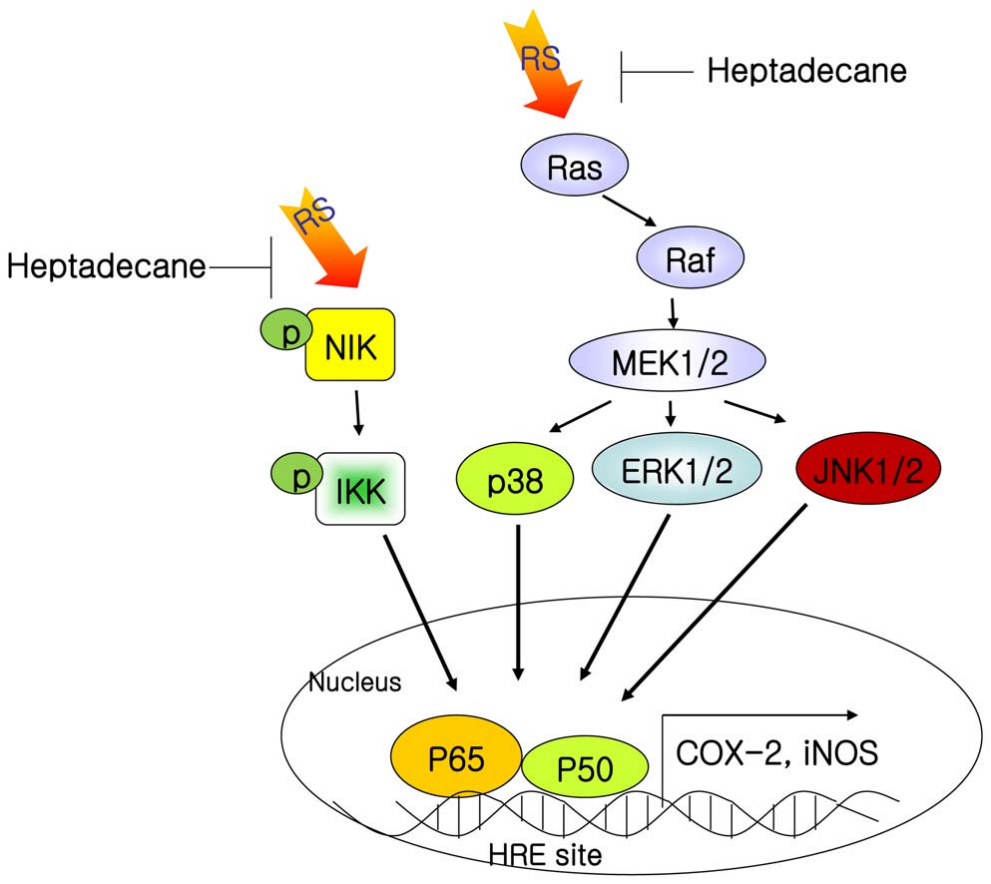
Possible mechanism of the effect of aging and heptadecane on the NIK/MAPKs/NF-kB pathway. RS, reactive species; NF-kB, nuclear factor kappa B; NIK, NF-kB-inducing kinase; IKK, IkB kinase; COX-2, cyclooxygenase-2.R.

To investigate crosslink redox modulation by the anti-oxidative action of heptadecane, we utilized the strong oxidant t-BHP to cause redox imbalance, and thus, mimic the aging environment [21], [36], and specific inhibitors of IKK and MAPKs to detect NF-kB-dependent COX-2 transcription [37], [38]. These experiments were designed to confirm that phosphorylation of the IKK or the MAPK pathway is induced by age-related oxidative stress and that these phosphorylations lead to NF-kB activation. We found that heptadecane significantly inhibited NIK/IKK and the activations of MAPKs and NF-kB (Fig. 8). Furthermore, the antiinflammatory effects of heptadecane were evident in oxidative stress-induced endothelial cells and in aged kidney tissues.

Summarizing, these results suggest that heptadecane is an important anti-inflammatory compound with potential therapeutic applications for the treatment of NF-kB-related or NF-kB dependent gene-related conditions in the elderly.

## Materials and Methods

### Animals

Young and aged specific pathogen-free (SPF) rats were obtained from Samtako (Osan, Korea) and fed a diet of the following composition: 21% soybean protein, 15% sucrose, 43.65% dextrin, 10% corn oil, 0.15% a-methionine, 0.2% choline chloride, 5% salt mix, 2% vitamin mix, and 3% Solka-Floc. All rats were allowed food *ad libitum* (AL) and were divided into two groups, namely, the AL-fed control group and the heptadecane-administrated group. For experiments using the oral administration of heptadecane, SPF male Sprague Dawley (SD) rats at 9 and 20 months of age were used as young and aged rats, respectively. To investigate the effects of heptadecane on NF-κB regulation during aging, heptadecane was ground and then mixed 0.01% or 0.02% in rat chow and fed to 20-month-old rats for 10 days. Considering that each animal consumed an average of between 2 and 4 mg of the drug daily, the cumulated dose per rat would be 20 to 40 mg/Kg. The animal protocol used in this study has been reviewed and approved by the Pusan National University-Institutional Animal Care and use Committee (PNU-IACUC) on their ethical procedures and scientific care.

The rats were sacrificed by decapitation and the kidneys were quickly removed and rinsed in iced-cold buffer [100 mM Tris, 1 mM EDTA, 0.2 mM phenylmethyl-sulfonylfluoride (PMSF), 1 µM pepstatin, 2 µM sodium orthovanadate (pH 7.4)]. The tissue was immediately frozen in liquid nitrogen and stored at −80°C. The kidney was selected for the study based on: its sensitivity to oxidative stress, vulnerability to aging, and the responsiveness to age-related inflammatory responses, all which make the kidney as one of most suitable organs of the choice [39], [40]. We are thankful to the Aging Tissue Bank funded by Korea Science and Engineering Foundation for supplying aged tissue.

### Reagents

Heptadecane and *tert*-butylhydroperoxide (t-BHP) were purchased from Sigma (Sigma Chemical, MO, USA), and 2′,7′-Dichlorofluorescin diacetate (DCFDA) from Molecular Probes (Eugene, OR, USA). Various primary antibodies and horseradish peroxide-conjugated donkey anti-rabbit antibody were obtained from Santa Cruz Biotechnology (Santa Cruz, CA, USA) and from Amersham (Amersham, Bucks, UK), respectively. Polyvinylidene difluoride (PVDF) membranes were obtained from Millipore (Bedford, MA, USA). The radionucleotide [^32^P]-ATP was obtained from Amersham (Bucks, UK). Specific kinase inhibitors were purchased from Calbiochem (Darmstadt, Germany). All other chemicals were from Sigma and were of the highest purity available. Western blotting detection reagents were obtained from Amersham Pharmacia Biotech (Bucks, UK). Antibodies against p65, p50, COX-2, iNOS, p-NIK, p-IKK, IKK, p-p38, p38, p-ERK, ERK, P-JNK1/2, p-Raf, Raf, p-IkB, IkB, Rac 1, b-actin, and Histone H1 were obtained from Santa Cruz Biotechnology (Santa Cruz, CA, USA). Anti-phospho MEK1/2 was from Cell Signaling (Cell Signaling Technology, MA, USA).

### Reactive Species Scavenging Activity

2,7-Dichlorodihydrofluorescin diacetate (DCFDA) is oxidized to fluorescent 2,7-dichlorofluorescein (DCF) by RS [41]. Utilizing menadione as the source of superoxide, a working solution of 25 µM DCFDA diluted from stock solution was placed on ice in the dark immediately prior to the study. The fluorescence intensity of DCFDA was measured for 30 min by microplate fluorescence using excitation and emission wavelengths of 485 and 535 nm, respectively.

### Tissue Preparation

The kidneys used in this study were taken from SPF male SD rats maintained in barrier facilities at Samtako (Osan, Korea). Complete descriptions of the housing, care, and feeding of animals have been reported previously [42]. Rats were sacrificed by decapitation, chests were opened, and kidneys were quickly excised. Kidneys were then immediately immersed in ice-cold isotonic saline and then in liquid nitrogen and stored at −80°C.

All tissues were homogenized in ice-cold homogenization solution [50 mM potassium phosphate buffer (pH 7.4), containing 1 mM EDTA, 1 mM p-aminobenzamidine, 1 µM pepstatin, 1 µM leupeptin, 1 µM aprotinin, 2 mM sodium orthovanadate, and 20 mM sodium fluoride] and centrifuged at 900 g at 4°C for 15 min. Supernatants were recentrifuged at 12,000 g at 4°C for 15 min and resulting pellets were regarded as cytosolic fractions.

For nuclear proteins, all solutions, tubes, and centrifuges were maintained at 0–4°C. Three rat kidney tissues were pooled for nuclear extract preparations, which were conducted as previously described [40]. The total protein concentrations of samples were measured using a protein assay reagent kit containing bicinchoninic acid (Sigma, USA).

### Intracellular Oxidative Stress and Toxicity in Cultured Cells

YPEN-1 cells (a rat prostatic endothelial cell line) were obtained from the ATCC (American Type Culture Collection, Rockville, MD, USA). Cells were cultured in Dulbecco’s Modified Eagle Media (DMEM) (Nissui Co., Tokyo) supplemented with 5% heat-inactivated (56°C for 30 min) fetal bovine serum (Gibco, Grand Island, NY).

To determine intracellular RS scavenging activity, YPEN-1 cells were seeded in a 96-well plate. After one day, the medium was changed to a fresh serum-free medium. The cells were then treated with or without heptadecane and pre-incubated for 1 hr. Cells were then treated with t-BHP (10 µM) for 30 min, media were replaced with fresh serum free medium, and H_2_DCFDA (f.c. 2.5 µM) was added DCF fluorescence intensities were measured every 5 min for 1 hr by microplate fluorescence using excitation and emission wavelengths of 485 and 535 nm, respectively. For visible detection of intracellular RS, YPEN-1 cells seeded in a 96-well plate were incubated with heptadecane and t-BHP, the medium was then removed, and DCFDA (5 µM) was added. Cells were observed under a fluorescence microscope at X120 (Axiovert 100; Zeiss, Germany).

Cell survival was quantified colorimetrically using 3-(4,5-dimethylthiazol-2-yl)-2,5-diphenyl-tetrazolium bromide (MTT, Sigma), which measures mitochondrial activity in viable cells. Briefly, cells were seeded at a density of 3×10^4^ per well in a 48-well plate and allowed to adhere overnight. Culture medium was then replaced with fresh serum-free DMEM. Cells were then pre incubated for 1 hr with heptadecane and treated with 10 µM t-BHP. MTT was freshly prepared at 5 mg/mL and a 0.5-mL aliquot of MTT stock solution was added to each well, and incubated at 37°C for 3 hr. Cells were then disrupted with solubilization solution (DMSO/absolute ethanol, 1∶1) and the formazan dye produced by viable cells was quantified using an ELISA microplate reader at 560 nm.

### Preparation of Cytosolic and Nuclear Extracts from Cultured Cells

Heptadecane and/or t-BHP treated YPEN-1 cells were washed with PBS and then 1 ml of ice-cold PBS was added. Pellets were harvested at 3,000 rpm at 4°C for 5 min, suspended in 10 mM Tris (pH 8.0) containing 1.5 mM MgCl_2_, 1 mM DTT, 0.1% NP-40, and protease inhibitors, and incubated on ice for 15 min. Nuclei were separated from cytosol by centrifugation at 12,000 rpm at 4°C for 15 min.

Supernatants were designated cytosolic fractions and pellets were resuspended in 10 mM Tris (pH 8.0) containing 50 mM KCl, 100 mM NaCl, and protease inhibitors, incubated on ice for 30 min, and centrifuged at 12,000 rpm at 4°C for 30 min. Supernatants were designated nuclear fractions.

### NF-kB-dependent Reporter Gene Assay

NF-kB activity was examined using a luciferase plasmid DNA, pTAL-NF-κB, which contains a specific binding sequence for NF-kB (BD Biosciences Clontech, CA, USA) [28]. Transfection was carried out using FuGENE 6 Reagent (Roche, Indianapolis, IN). Briefly, 2×10^4^ cells per each well were seeded in 48-well plates. When cultured cells reached about 50% confluence, they were treated with 0.1 µg DNA/0.2 µl FuGENE 6 complex in a total volume of 500 µl made up with normal media (containing 5% serum) for 48 hr. After replacing media with serum-free media 0.2 µM of t-BHP was added, and treatments in several doses of heptadecane were performed 1 hr previously. After additional incubation for 6 hr, cells were washed with PBS and the Steady-Glo Luciferase Assay System (Promega, Madison, WI, USA) to the plate. Luciferase activity was measured using a luminometer (GENious, TECAN, Salzburg, Austria).

### Protein Measurements by Western Blotting

To investigate changes in the expressions of different proteins, we used Western blot experiments to examine cytosolic and nuclear fractions of kidney tissues. Samples were boiled for 5 min with a gel loading buffer (125 mM Tris–HCl, 4% SDS, 10% 2-mercaptoethanol, pH 6.8, and 0.2% bromophenol blue) at a ratio of 1∶1. Total protein equivalents for each sample were separated on 8–15% SDS–polyacrylamide mini-gels using a Laemmli buffer system. Samples were then transferred to polyvinylidene difluoride (PVDF) membranes at 100 V for 1.5 h. Blots were blocked using 1–5% non-fat milk in 10 mM Tris, pH 7.5, 100 mM NaCl, and 0.1% Tween-20 at room temperature for 1 hr, and then incubated with specific primary antibody followed by a secondary antibody (horseradish peroxidase-conjugated donkey anti-rabbit antibody; Amersham, Bucks, UK, 1∶5,000) at 25°C for 2 hr respectively. Antibody labeling was detected by enhanced chemiluminescence (ECL) (Amersham) as per the manufacturer’s instructions and blots were then exposed to Hyperfilm (Amersham).

### Electrophoretic Mobility Shift Assay (EMSA)

EMSA was used to characterize the binding activities of NF-kB transcription factors in nuclear extracts [43]. The NF-κkB oligonucleotide sequence used was 5′-GAGAGGCAAGGGGATTCCCTTAGTTAGGA-3′. Protein-DNA binding assays were performed with 20 µg of nuclear protein. Unspecific binding was blocked using 1 µg of poly(dI-dC)•poly(dI-dC). The binding medium contained 5% glycerol, 1% NP40, 1 mM MgCl2, 50 mM NaCl, 0.5 mM EDTA, 2 mM DTT, and 10 mM Tris/HCl, pH 7.5. In each reaction, 20,000 cpm of a radiolabeled probe was included.

Samples were incubated at room temperature for 20 min, and nuclear proteins labeled with ^32^P-labeled oligonucleotide complex were separated from free ^32^P-labeled oligonucleotide by electrophoresis through a 5% native polyacrylamide gel in a running buffer containing 50 mM Tris, pH 8.0, 45 mM borate and 0.5 mM EDTA. After separation has been achieved, gels were vacuum dried for autoradiography and exposed to Fuji X-ray film for 1–2 days at −80°C.

### Immunohistochemistry

The paraffin sections from rat renal tissues were prepared [44] for immunohistochemical assays following the protocol. The sections were deparaffinized in xylene and dehydrated through graded ethanol, and washed thrice with PBS (0.02 mmol/L, pH 7.4) for 3 min. The sections were incubated with 3% H_2_O_2_ for 10 min. After rinsing for 3 min×3 with PBS, the sections were treated to boil in a microwave oven twice in 10 mM sodium citrate buffer (pH 6.0), then kept in 5% BSA for 30 min after rinsing for 3 min×3 with PBS, followed by incubation with a polyclonal antibody to NF-κB (Sc-109, santacruz, 1∶100 dilution) overnight at 4°C. The sections were rinsed for 2 min×3 with PBS before incubation with broad spectrum antibody for NF-κB, for 20 min at 20°C, and incubated with HRP-streptavidin for 20 min (Invitrogen, 859943), and then rinsed for another 10 min×2 with PBS before reaction with DAB solution for 15 min. Finally, the sections were counterstained with hematoxylin and then enveloped with gelatin for Moticam Pro 205A microscopi exmanination.

### Statistical Analysis

One-factor ANOVA was used for the statistical analysis. Statistical significance was accepted for p values <0.05.
